# A Novel Mouse Model of Soft-Tissue Infection Using Bioluminescence Imaging Allows Noninvasive, Real-Time Monitoring of Bacterial Growth

**DOI:** 10.1371/journal.pone.0106367

**Published:** 2014-09-03

**Authors:** Kenji Yoshioka, Ken Ishii, Tetsuya Kuramoto, Shigenori Nagai, Haruki Funao, Hiroko Ishihama, Yuta Shiono, Aya Sasaki, Mamoru Aizawa, Yasunori Okada, Shigeo Koyasu, Yoshiaki Toyama, Morio Matsumoto

**Affiliations:** 1 Department of Orthopedic Surgery, School of Medicine, Keio University, Shinjuku, Tokyo, Japan; 2 Department of Microbiology and Immunology, School of Medicine, Keio University, Shinjuku, Tokyo, Japan; 3 Department of Pathology, School of Medicine, Keio University, Shinjuku, Tokyo, Japan; 4 Department of Applied Chemistry, School of Science and Technology, Meiji University, Kawasaki, Kanagawa, Japan; 5 Kanagawa Academy of Science and Technology (KAST), Kawasaki, Kanagawa, Japan; 6 Core Research for Evolutional Science and Technology (CREST), Japan Science and Technology Agency (JST), Tokyo, Japan; 7 Department of Molecular Immunology, Graduate School of Medical and Dental Sciences, Tokyo Medical and Dental University, Tokyo, Japan; 8 Laboratory for Immune Cell System, RIKEN Center for Integrative Medical Sciences (IMS), Yokohama, Kanagawa, Japan; The Scripps Research Institute and Sorrento Therapeutics, Inc., United States of America

## Abstract

Musculoskeletal infections, including surgical-site and implant-associated infections, often cause progressive inflammation and destroy areas of the soft tissue. Treating infections, especially those caused by multi-antibiotic resistant bacteria such as methicillin-resistant *Staphylococcus aureus* (MRSA) remains a challenge. Although there are a few animal models that enable the quantitative evaluation of infection in soft tissues, these models are not always reproducible or sustainable. Here, we successfully established a real-time, *in vivo*, quantitative mouse model of soft-tissue infection in the superficial gluteus muscle (SGM) using bioluminescence imaging. A bioluminescent strain of MRSA was inoculated into the SGM of BALB/c adult male mice, followed by sequential measurement of bacterial photon intensity and serological and histological analyses of the mice. The mean photon intensity in the mice peaked immediately after inoculation and remained stable until day 28. The serum levels of interleukin-6, interleukin-1 and C-reactive protein at 12 hours after inoculation were significantly higher than those prior to inoculation, and the C-reactive protein remained significantly elevated until day 21. Histological analyses showed marked neutrophil infiltration and abscesses containing necrotic and fibrous tissues in the SGM. With this SGM mouse model, we successfully visualized and quantified stable bacterial growth over an extended period of time with bioluminescence imaging, which allowed us to monitor the process of infection without euthanizing the experimental animals. This model is applicable to *in vivo* evaluations of the long-term efficacy of novel antibiotics or antibacterial implants.

## Introduction

Musculoskeletal infection is a serious problem in orthopedic and dermatologic medicine. It has become difficult to manage infections conservatively with antibiotics because of *methicillin-resistant Staphylococcus aureus* (MRSA) and other multi-drug-resistant pathogens, implant-associated infections, and immune-system compromise in elderly and other patients. To understand the pathophysiology of musculoskeletal infections, experimental studies using animal infection models can be very valuable. A number of animal models of musculoskeletal infection have been reported, most of which simulate osteomyelitis [1]–[6]. Although a few animal models of soft-tissue infection have been reported [7]–[9], these models are not always reproducible or sustainable due to the leakage and spread of bacteria from the inoculated tissues. Furthermore, sequential analyses of the extent of musculoskeletal infection and number of bacteria in these models require sacrifice of the animals at each analysis time point. Such an invasive approach makes it difficult to detect the real kinetics and growth of the bacteria.

Recent advances in optical imaging such as bioluminescence imaging (BLI) and fluorescence reflectance imaging (FRI) have made it possible to monitor implanted cells or specific gene expressions *in vivo* in real-time without euthanizing the animal. This approach has proven useful in various fields of study, including regenerative medicine [10], [11], oncology [12], endocrine disruptors [13], metabolism [14], hematopoietic cells [15], immunity [16], [17], and infections [3]. A number of studies have demonstrated the advantages of *in vivo* BLI for real-time monitoring of bacterial infections and treatments in various tissues, including the flank [4], [18], thigh [1], [8], subcutaneous area [9], heart [19], lung [20], bone [2], and joint [21]. In BLI, bioluminescent bacteria inoculated into tissue emit a constant signal that can be detected through the tissue of a living animal using an ultrasensitive, cooled charge-coupled device (CCD) camera. Studies show that *in vivo* BLI is significantly faster and more sensitive than standard techniques for the real-time monitoring of bacterial infections and their treatment [2], [3], [9], [11], [22], [23]. This technology enables quantitative and spatial data to be acquired from defined areas of the animal. We recently established a quantitative, noninvasive, and reliable mouse femur osteomyelitis model using BLI [2]. This model can be used to clarify the pathology and kinetics of osteomyelitis and to evaluate *in vivo* novel therapeutic strategies, including newly developed antibiotics or bacterium-resistant implants, before conducting studies in larger animals or in human subjects [7], [9], [14], [19]. However, it is difficult to evaluate the antibacterial effect of bulky materials such as artificial bone or ceramic implants, because the femur is so small. Thus, there is a need for infection models that allow the bacteria to be confined within a relatively large inoculated tissue and enable the long-term evaluation of implants or other treatments.

In the present study, we chose the superficial gluteus muscle (SGM), which is the largest mouse muscle with a thick fascia, as the infection site, and sought to establish a real-time, quantitative, and reproducible mouse model of soft-tissue infection for tracking bacterial infection over extended periods by BLI.

## Materials and Methods

### Bioluminescent bacteria

A bioluminescent MRSA strain (Xen-31) purchased from Caliper LS Co. (Hopkinton, MA) was maintained in Luria Bertani medium (Sigma-Aldrich Co., St. Louis, MO) at 37°C, under ambient aeration with gentle agitation. The bacteria were grown selectively on medium containing 200 µg/ml kanamycin. MRSA Xen-31, derived from the parental strain American Type Culture Collection (ATCC) 33591, has a stable copy of a modified *Photorhabdus luminescens luxABCDE* operon, which encodes the enzymes responsible for the luminescent reaction. No additional substrates were necessary for bioluminescence; the bacteria constitutively emitted a bioluminescent signal within the living organism. Bacteria samples were frozen and stocked at −80°C, and were thawed at 4°C for one hour before use. Bacterial viability was typically maintained at 4°C for approximately 5 hours after thawing.

### Mouse model of SGM soft-tissue infection

This study used twelve BALB/c adult male mice (12 weeks old, 20–25 g) obtained from Sankyo Labo Service (Shizuoka, Japan). The mice were maintained in our animal facility under specific pathogen-free conditions. To inject MRSA into the SGM, the mouse was anesthetized with an intraperitoneal injection of 50 mg/kg of pentobarbital, the left hip was widely sterilized with povidone iodine, and the mouse was placed prone on the surgical bed. A 22-gauge Hamilton syringe was used to inject 1.5×10^8^ colony-forming units (CFU) of MRSA in 5 µl of medium into the left SGM. The SGM is easily detected through the skin as a thick muscle between the lumbar spine and the proximal of the femur. The animal was placed on a heating pad and carefully monitored through recovery. The animal was judged to have recovered from the anesthesia when it demonstrated spontaneous forelimb movement and a normal heartbeat, and was able to drink water.

To measure and analyze the bacterial bioluminescent signals by BLI, the mice were anesthetized by inhaled aerosolized isoflurane mixed with oxygen, placed in the prone position, and imaged for 5 minutes.

This study was performed in strict accordance with recommendations in the Guide for the Care and Use of Laboratory Animals of the National Institutes of Health. The protocol was approved by the Keio University Committee on the Ethics of Animal Experiments (Permit Number: 09108-[3]), and all experiments were approved by the Animal Care and Use Committee of Keio University. All surgery was performed under sodium pentobarbital anesthesia, and all efforts were made to minimize suffering.

### Optical imaging (BLI and FRI)

A Caliper LS-IVIS Lumina (Summit Pharmaceuticals International Co., Tokyo, Japan) cooled CCD optical macroscopic imaging system was used for BLI and FRI. Photons emitted by the bioluminescent bacteria and by a fluorescent inflammation probe were captured by BLI and FRI, respectively. Photon signals were converted to false-color photon-count images, and were quantified with Living Image software version 3.0 (Caliper LS Co., Hopkinton, MA). The bacterial photon intensity (PI) was expressed as photon flux, in units of photons/sec/cm^2^/steradian. To assess bacterial luminescence using BLI, we first examined whether the number of bacteria correlated with the bacterial PI both *in vitro* and *in vivo*. To analyze the course of the infection over time *in vivo*, the bacterial PI in a region of interest (ROI) of the left SGM was measured immediately after the operation and on days 1, 3, 7, 14, 21, and 28. To detect the area of inflammation around the infection site, we used a Cy5.5-conjugated inflammation probe (Katayama Chemical Ltd., Osaka, Japan) that binds the adhesion molecule E-selectin, which is expressed in vascular endothelial cells [24]. Neutrophils detect and bind E-selectin, which normally has a low basal expression. Therefore, the inflammation area observed by FRI with this probe shows the spread of the host neutrophils. On day 10, the Cy5.5-conjugated inflammation probe was injected via the tail vein, and the inflammation surrounding the bacterial inoculation area was visualized by FRI. The luminescence or fluorescence was quantified within defined ROIs on bacteria plates or in inoculated areas.

### Bacterial quantification

**Figure 1:**
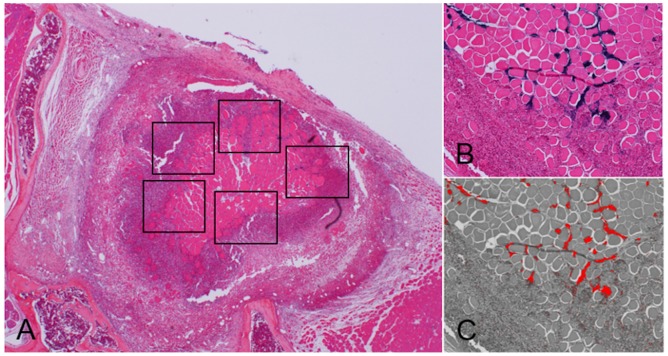
Bacterial quantification. To evaluate the correlation between the bacterial PI and the number of bacteria in the infectious process *in vivo*, the positive area of gram-stained MRSA on the each tissue section from the different time-point animals (3, 7, and 28 days after inoculation) were calculated. Six axial sections which cover whole abscess in SGM were obtained from each animal. The total numbers of pixels in gram-positive area (blue stains) were measured in five fields from six sections (A). Image obtained from software (B) shows that gram-positive blue area has been replaced to red pixel dots (C).

To analyze the correlation between the luminescence signal and the number of bacteria during the infectious process *in vivo*, the positive area of gram-stained MRSA on the each tissue section from the different time-point animals (3, 7, and 28 days after inoculation) were calculated. Six axial sections which cover whole abscess in SGM were obtained from each animal for measurement. Then, five fields in the abscess were selected in each axial section (Figure 1A). The total numbers of pixels in gram-positive area were measured in five fields from six sections using SigmaScan Pro Image software (SPSS Inc., Chicago, IL) (Figure 1B and C). The relationship between total pixels of total gram-positive area and its PI was investigated.

### Serological analysis

Blood samples (approximately 150 µl) were collected from the tail vein of each animal before inoculation (day 0) and 0.5, 1, 3, 7, 14, and 21 days after inoculation. The samples were incubated at 37°C for 30 minutes and then centrifuged at 14,000 rpm for 15 minutes to obtain the serum. To measure inflammatory cytokines, the serum levels of interleukin-6 (IL-6), interleukin-1 (IL-1β), and C-reactive protein (CRP) were measured with enzyme-linked immunosorbent assay (ELISA) kits (R&D Systems, Kamiya Biomedical Company), according to the manufacturer's instructions.

### Histological analysis and bacterial quantification

Samples were collected on 3, 7, 28, and 42 days after inoculation; the mice were sacrificed and the pelvis was removed along with the SGM. The samples were fixed in 4% paraformaldehyde, demineralized with a 10% ethylenediaminetetraacetic acid solution, embedded in paraffin, cut into 5- µm-thick axial sections, and stained with hematoxylin/eosin and gram stains.

### Statistical analysis

Correlations between the number of bacterial CFU and the bacterial PI *in vitro* and *in vivo* were analyzed by linear regression. Changes in bacterial PI were analyzed with one-way ANOVA. The serum levels of IL-6, IL-1β, and CRP at various time points were analyzed with Student's *t* test. Statistical analyses were performed with SPSS II software (IBM-SPSS, Tokyo, Japan), and a probability value less than 0.05 was considered significant.

## Results

### Correlation between the number of bacteria and bacterial PI *in vitro*

**Figure 2:**
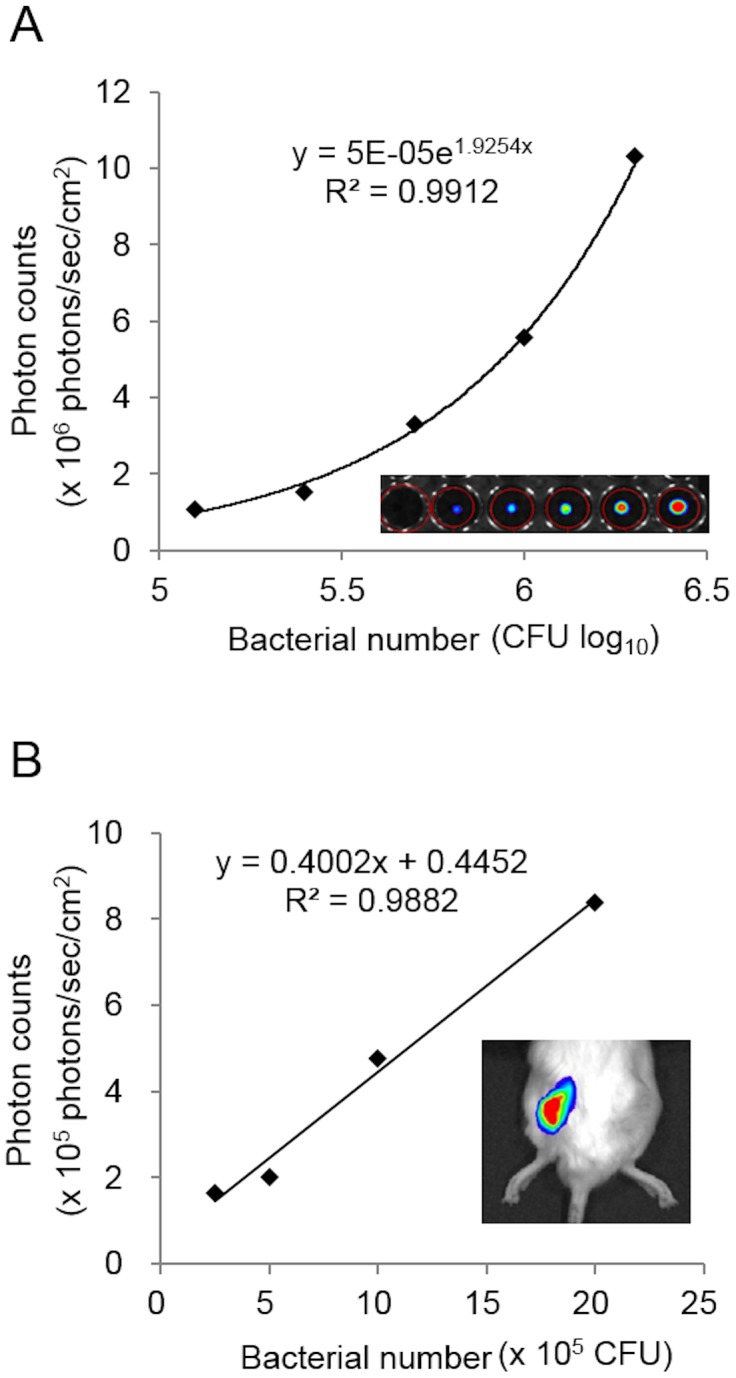
Correlation between the number of bacteria and bacterial PI *in vitro* and *in vivo*. BLI sensitivity was assessed using a CCD-based macroscopic detector to quantify the bacterial photon intensity (PI  =  photons/sec/cm^2^/steradian) for various numbers of bacteria (1.25×10^5^ to 2.0×10^6^ CFU per well). (A) *In vitro* study: the bacterial PI from colonies of bioluminescent MRSA was significantly correlated with the number of bacterial CFUs (*R* ^2^ = 0.9912). (B) *In vivo* study: various numbers of bacteria (1.25×10^5^–2.0×10^6^ CFU per inoculation) were inoculated into the SGM, and bioluminescence in the region of interest (ROI) was monitored by BLI. There was a significant correlation between the number of inoculated bacteria and the bacterial PI *in vivo* (*R* ^2^ = 0.9882).

A single colony of the bioluminescent Xen-31 MRSA strain, cultured in Luria Bertani medium, emitted sufficient bioluminescence to be detected by BLI. To examine the BLI sensitivity, we used a CCD-based macroscopic detector in BLI to measure the PI of bacterial samples *in vitro* at 1.25×10^5^ to 2.0×10^6^ CFU per well. This quantitative bioluminescent analysis demonstrated that the bacterial PI was directly proportional to the number of bacterial CFUs *in vitro* (*R* ^2^ = 0.9912) (Figure 2A). To confirm that the luminescent signals were naturally emitted only by live bacteria, MRSA colonies were fixed with 4% paraformaldehyde and visualized by BLI. No signal was detected from the fixed bacteria (data not shown).

### Correlation between the number of bacteria and bacterial PI *in vivo*

To determine whether the number of bacteria inoculated correlated with the bacterial PI *in vivo*, 1.25×10^5^ to 2.0×10^6^ CFU of MRSA in 5 µl of medium were inoculated into the mouse SGM and were measured by BLI *in vivo*. Analysis of the bioluminescence showed that the bacterial PI was directly proportional to the number of bacterial CFUs *in vivo* (*R* ^2^ = 0.9882) (Figure 2B).

### Bacterial PI over time in the mouse SGM model

**Figure 3:**
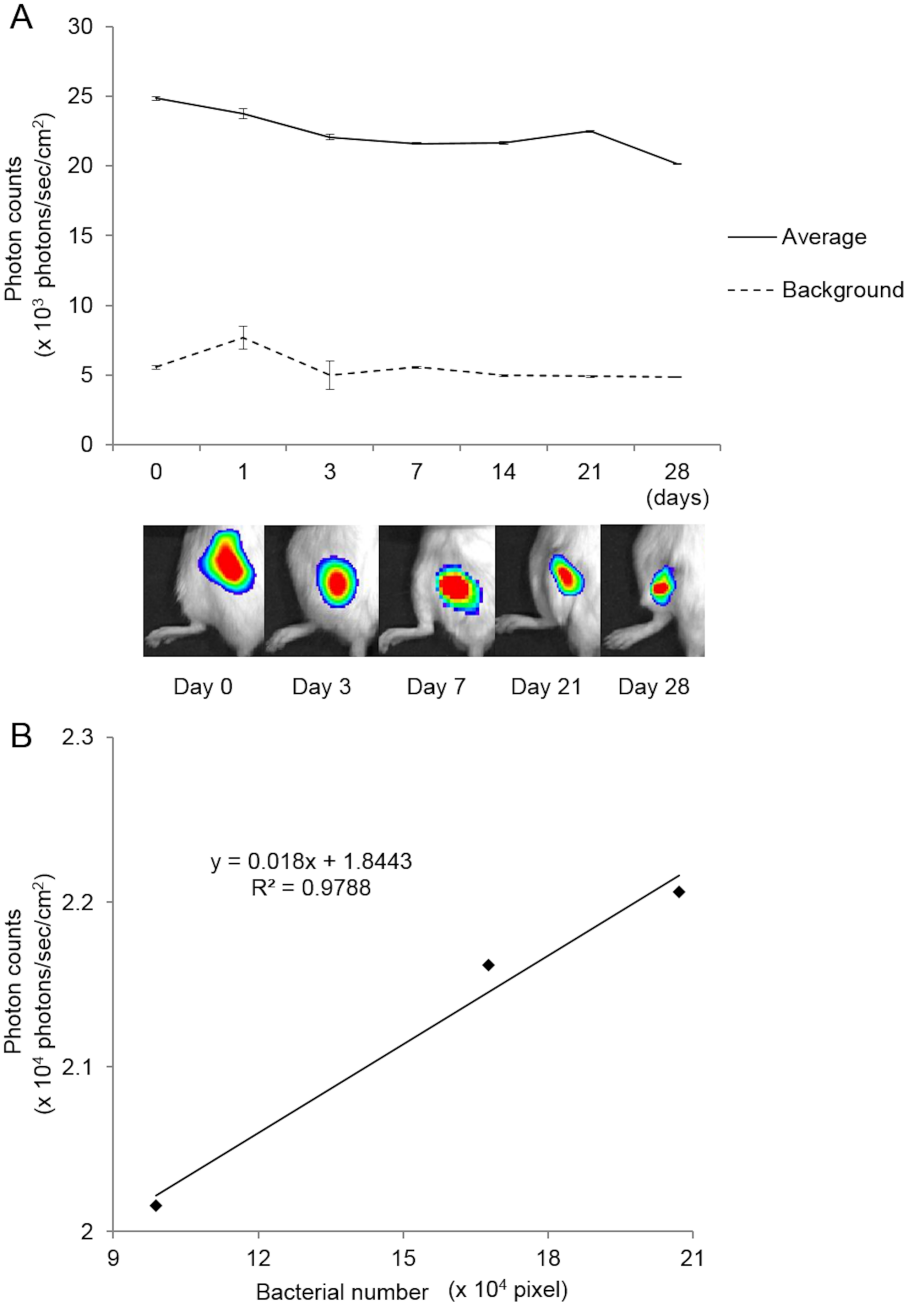
Changes in bacterial PI in the mouse SGM model and correlation between the number of inoculated bacteria and bacterial PI in the infection process. We inoculated MRSA Xen31 (3×10^7^ CFU/µl) in 5µl of medium into the SGM, and measured the bacterial PI in the ROI immediately after inoculation, and on days 1, 3, 7, 14, 21, and 28 (N = 6). (A) The mean bacterial PI in the SGM peaked immediately after inoculation (2.486×10^4^ PI) and remained high for at least 28 days (2.016×10^4^ PI). Means and SEM are shown. (B) There was a significant correlation between the pixels number of gram-positive area and the bacterial PI during 28 days *in vivo* (*R^2^* = 0.9788).

After inoculating MRSA (1.5×10^8^ CFU) in 5 µl of medium into the left SGM, stable luminescent signals were detected in all of the animals. Sequential analyses of the bacterial luminescence showed that the mean bacterial PI in the SGM peaked immediately after inoculation (2.486×10^4^ PI) and remained high for at least 28 days (2.016×10^4^ PI) (Figure 3A). The mean background signal was 5.309×10^3^ PI. The mean bacterial PI was significantly higher than the background PI at all time points. Notably, strong bacterial bioluminescence was detected only at the injection site in the SGM, without any bioluminescence in the right SGM or in the subcutaneous space in any of the animals, indicating that the bacteria stayed within the SGM. Hair loss was observed at the injection site, typically about 7 days after inoculation, in all animals.

### Correlation between the number of inoculated bacteria and bacterial PI during the process of infection

To assess whether the number of bacteria inoculated correlated with the bacterial PI during the process of infection, bacterial PI and gram-positive area (pixels counts) from the SGM tissue sections were measured. There was a significant correlation between the pixels number of gram-positive area and the bacterial PI *in vivo* (*R^2^* = 0.9788) (Figure 3B).

### Dual optical imaging (BLI and FRI)

**Figure 4:**
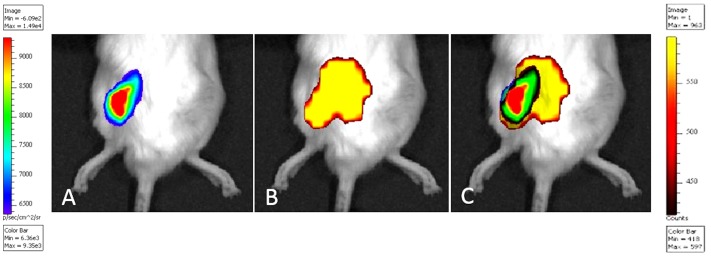
Dual optical imaging in the mouse SGM model. (A) Bioluminescence and (B) fluorescence images in the same animal, 10 days after bacterial inoculation, are shown. (A) MRSA inoculated into the left SGM was sufficient to produce an observable bioluminescence signal. (B) A Cy5.5-conjugated inflammation probe accumulated at the inoculation site only 5 minutes after it was injected into the tail vein, and was monitored for over 2 weeks in the same animal. (C) In the merged image, the accumulation of the inflammation probe (yellow) appeared as an area of fluorescence that completely covered the entire region of bacterial bioluminescence signal (multi-color).

To detect the extent of inflammation surrounding the inoculation site, a Cy5.5- conjugated inflammation probe was injected via the tail vein 10 days after inoculation. Interestingly, the Cy5.5-conjugated probe accumulated at the inoculation site only 5 minutes after it was injected into the tail vein. Dual optical imaging with BLI and FRI revealed that the fluorescent inflammation probe accumulated in an area that completely covered the area of bacterial bioluminescence (Figure 4).

### Serological evaluation

**Figure 5:**
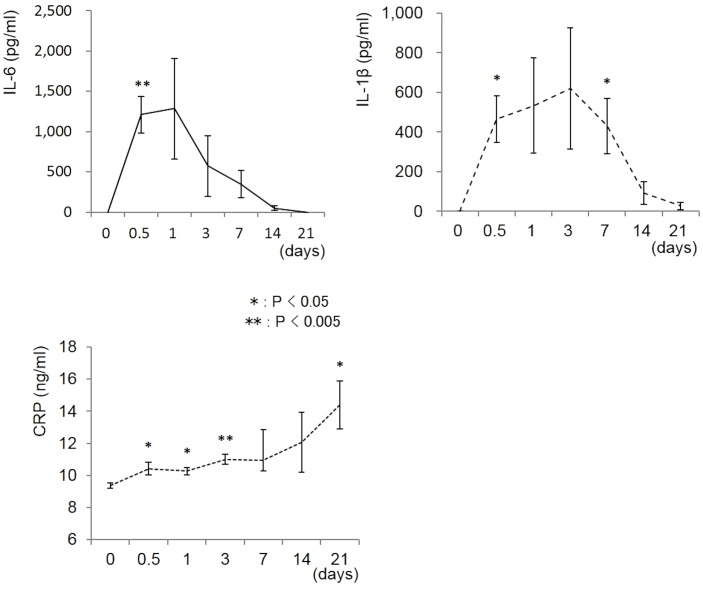
Serological data. Mean serum levels of IL-6, IL-1β, and CRP in MRSA-inoculated animals at various time points are shown (N = 4 each). Compared to the pre-inoculation level, the IL-6 level was significantly higher 12 hours after inoculation (*P*<0.05). The IL-6 level gradually decreased, becoming normal by day 21. IL-1β was significantly elevated after 12 hours, and remained high for 7 days (P<0.05). CRP was significantly higher than the pre-inoculation level 0.5, 1, 3, and 21 days after inoculation (*P*<0.05). The means and SEM are shown.

Serological analyses showed elevated mean serum levels of IL-6, IL-1β, and CRP in the MRSA-infected animals during the early phases of infection (Figure 5). Compared to the pre-inoculation level, IL-6 was significantly elevated 12 hours after inoculation (*P*<0.005), after which it gradually decreased until reaching the pre-inoculation level on day 21. IL-1β was significantly elevated 12 hours after inoculation, and remained high for 7 days (*P*<0.05). The CRP level was significantly elevated at 0.5, 1, 3, and 21 days compared to the level prior to inoculation (*P*<0.05, 0.05, 0.005, and 0.05, respectively).

### Histological analysis

**Figure 6:**
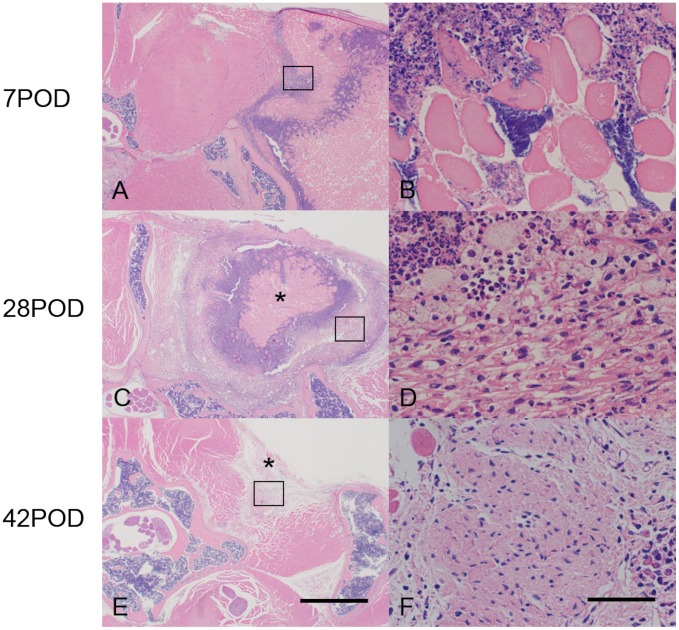
Histological findings. Changes in histology over time in the SGM from infected mice. Axial sections of the infected SGM were prepared at 7 (A and B), 28 (C and D), and 42 (E and F) days after bacterial inoculation and stained with hematoxylin and eosin. Lower images: magnified views of the boxed regions in the upper images. On day 7, marked neutrophil infiltration and muscle destruction were detected at the injection sites. On day 28, an abscess that contained necrotic materials (asterisk) and covered by fibrous tissue was observed in the SGM. On day 42, scaring tissue (asterisk) observed in the subcutaneous tissue. Bars  = 200 µm (A, C, E), 10 µm (B, D, F).

Histology showed marked neutrophil infiltration into the inoculation site on days 3 and 7 (Figure 6A and B for day 7; data not shown for day 3). On day 28, abscesses, which contained necrotic materials in the center and covered by fibrous capsule-like structure, were seen in the SGM (Figure 6C and D). On day 42, the abscesses reduced and scaring tissues were observed in the subcutaneous tissue (Figure 6E and F).

## Discussion

Newly developed optical imaging techniques such as BLI and FRI enable the longitudinal real-time monitoring of bacterial growth in living animals, throughout the course of infection in various organs [2]–[6], [19], [23]. Models of bone or joint infection are commonly used to study musculoskeletal infections [2], [5], [21], [25]. A few animal models have been reported for soft tissue and skin infection, including the flank [4], [18], thigh [1], [8], and subcutaneous areas [9]. However, these soft-tissue models have some disadvantages; these tissues lack a strong barrier to confine the inoculated bacteria, or there is insufficient tissue volume for bacterial inoculation. Thus, these models are not always reproducible or sustainable because of the leakage and spread of the bacteria from the inoculated tissues. Indeed, in previous studies the bacterial growth was observed for periods ranging from only 12 hours to a maximum of 7 days [3], [8], [18], [26]. In models of osteomyelitis and joint infection, skin ulcers resulting from bacterial leakage from the inoculation site to surrounding tissues are often observed, making such models unstable and poorly reproducible. We chose the SGM, the largest mouse muscle, as an inoculation site in our present study, because its thick fascia confines the growing bacteria within a large muscle volume, making it suitable for observation over longer periods of time. With these advantages, we have successfully established a stable, reproducible animal model of soft-tissue infection. Our data showed stable bacterial bioluminescence, observable by BLI, at the inoculated region for at least 4 weeks. The bacteria and abscesses were confined to the left SGM even 28 days after inoculation, and non-SGM tissue specimens from the inoculated animals did not show any leakage of the bacteria.

Another reason for the relatively short observation periods with previous animal models is the use of the SH1000 *S*. *aureus* bioluminescent strain, in which the *lux* genes are contained in a plasmid [21] that is only stable for the first 3 days of broth culture, after which it is difficult to estimate the number of bacteria from the bioluminescent signals [21]. Thus, such a model represents only acute, but not chronic, infections. In our model, the *lux* genes were inserted into the *S. aureus* chromosome, so the bioluminescent signals were maintained over a long period. Thus, this model is useful to study pathophysiologies in both acute and chronic soft-tissue infection.

Our model used a simple inoculation technique. The SGM is easy to locate through the skin due to its size, which is large enough to accommodate an inoculation up to 10 µl in volume (data not shown). Interestingly, hair loss was observed in the skin over the infected SGM. The relationship between hair loss and infection was assumed that alkaline protease secreted by MRSA provokes a dehairing at the infection area [27], [28]. It was suggested that a macroscopic dehairing lesion reveals the occurrence of infection in the SGM.

To our knowledge, this is the first study to demonstrate a real-time, quantitative, reproducible soft-tissue infection mouse model that allows bacterial growth to be observed over a long period of time.

Our serological analysis showed that the serum levels of IL-6, IL-1β, and CRP increased immediately after the inoculation. IL-6 and IL -1β disappeared from the serum at 14 days, while the CRP level was maintained for at least 21 days. Pathological findings generally showed abscess formation beginning 14 days after the bacterial inoculation. Bacteria are encapsulated in the abscesses to prevent leakage from the infection site. Our data suggest that the serum levels of inflammatory cytokines decreased due to the isolation of the bacteria from the host tissues, while CRP remained high in the chronic phase. Since changes in the histology and the serum levels of inflammatory cytokines over time were similar to those observed in human patients [29], [30], our model may be useful for understanding the pathophysiology of acute and chronic inflammatory processes in human soft-tissue infection.

In the present study, we were able to indirectly visualize and measure the extent of host neutrophils in the infected site by fluorescence optical imaging. The distinct wavelengths of bioluminescence (490 nm) and Cy5.5-fluorescence (Ex. 680 nm, Em. 720 nm) allowed us to measure bacteria growth and the extent of neutrophil infiltration sequentially and longitudinally in the same SGM model animal, using dual optical imaging with BLI and FRI. The Cy5.5-conjugated inflammation probe stably binds the adhesion molecule E-selectin, which is expressed in vascular endothelial cells [24]. Neutrophils generally detect and bind E-selectin expressed on the vascular surface. Therefore, the Cy5.5-fluorescent area observed by FRI reflects the spread of host neutrophils. This novel dual optical imaging system using BLI and FRI can be used to clarify the pathology and kinetics of soft-tissue infection and to accurately evaluate novel *in vivo* therapeutic strategies, including the development of new antibiotics and bacterium-resistant implants, before performing studies in larger animals and human subjects.

In conclusion, we have successfully visualized and quantified bacterial growth in a mouse soft-tissue infection model using *in vivo* BLI. We were able to monitor the infectious process throughout the course of the disease, in both the acute and chronic phases, without sacrificing the animals. This animal model may be a powerful tool for evaluating the pathophysiology of infection and the efficacy of new antibacterial drugs and implants.
